# A Code-Conforming Computer Vision Framework for Visual Inspection of Reinforced and Prestressed Concrete Bridges

**DOI:** 10.3390/s26041242

**Published:** 2026-02-14

**Authors:** Giuseppe Santarsiero, Valentina Picciano, Nicola Ventricelli, Angelo Masi

**Affiliations:** Department of Engineering, University of Basilicata, 85100 Potenza, Italy; valentina.picciano@unibas.it (V.P.); angelo.masi@unibas.it (A.M.)

**Keywords:** computer vision, semantic segmentation, bridge inspection, damage detection, YOLO, code conforming

## Abstract

The assessment of structural degradation in reinforced concrete bridges is a crucial task for infrastructure maintenance and safety. Traditional inspection methods are often time-consuming, dependent on expert interpretation and weather conditions. This study explores the potential of artificial intelligence to support inspectors in the detection of typical deterioration patterns in reinforced (RC) and prestressed concrete (PRC) bridges, developing the VIADUCT (Visual Inspection and Automated Damage Understanding by Computer vision Techniques) software tool. Unlike previous studies, focusing only on a limited variety of possible defects (e.g., cracks, water stains), this study aims to train a deep learning model to be able to recognise a larger range of defects, such as those foreseen by the current Italian code for the assessment of existing bridges. The methodology relies on the YOLOv8n object detection model, which was trained, validated, and tested using a dataset including 1045 either wide-angle or detailed photographs taken during routine inspections. With these kinds of images being challenging for object detection algorithms (they include large parts of the background), multimodal attention mechanisms were implemented in the Graphical User Interface (GUI) through the semantic segmentation of the bridge surface using both the SAM and the U-Net model, as well as a tile reduction approach. These attention mechanisms allow the object detection model to focus on the relevant portions of the image (i.e., the bridge), while suppressing background information. Despite the limitation of the small size dataset used for training, results showed promising detection capabilities and precision. Furthermore, VIADUCT is ready to accept and use newer and more efficient versions of the object detection model, as soon as they become available.

## 1. Introduction

Bridge infrastructure plays a critical role in ensuring the connectivity and functionality of transport networks. In Italy, the morphological and orographic features of the territory have led to the widespread presence of viaducts, many of which are now exhibiting signs of advanced ageing and degradation. A large portion of these structures was built in the post-war period using reinforced and prestressed concrete, and many have already exceeded their conventional design service life. In the absence of timely and systematic maintenance actions, these structures risk failing to meet current safety standards. This issue has gathered increasing public and political attention, particularly after catastrophic failures such as the collapse of the Morandi Bridge in Genoa in 2018 [1], which highlighted the urgent need for robust inspection and maintenance practices across the national bridge network.

The Italian Ministry of Infrastructure responded to this need by publishing the 2020 “Guidelines for Risk Classification, Safety Assessment and Monitoring of Existing Bridges” [2]. These guidelines propose a multilevel approach for managing bridge infrastructure, from large-scale qualitative risk classification to detailed safety verification procedures [3]. Central to this framework is the assignment of a Class of Attention (CdA), which is a synthetic index of structural concern based on factors such as structural-founding risk, seismic risk, landslide risk, and hydraulic risk. The process begins with visual inspections, which constitute the fundamental instrument for assessing the condition of bridge components and determining the associated vulnerabilities. The outcome of these inspections directly influences the prioritisation strategies for maintenance, monitoring [4] and repair interventions.

However, visual inspections are not without limitations. Inspectors must physically access and examine all relevant structural surfaces, which is not always feasible due to accessibility constraints, operational disruptions, or the geometry of the structures and the weather conditions. Moreover, visual inspections are inherently subjective, leading to inconsistencies between reports produced at different times or by different inspectors. These drawbacks reduce the repeatability and reliability of condition assessments and hinder the standardisation required for data-driven infrastructure management. In this context, the application of artificial intelligence (AI), and, in particular, deep learning (DL), offers a promising opportunity to support and enhance current inspection procedures [5].

Recent years have witnessed rapid advancements in AI, driven by the availability of powerful computational resources and the proliferation of large datasets. Techniques such as computer vision (CV), convolutional neural networks (CNNs), and object detection frameworks have already demonstrated their value in automating tasks traditionally reliant on human perception. In civil engineering, DL has been applied to defect detection in pavements, crack identification in buildings, and post-earthquake damage assessment in structures. In the field of bridge inspection, several studies have proposed the use of YOLO (You Only Look Once) models [6] for the real-time recognition of surface defects or classification of bridge barriers [7], with encouraging results (e.g., [8]).

Nevertheless, there is still a lack of consensus regarding the standardisation of AI methodologies for use in practical bridge management systems. One of the main challenges is the preparation of high-quality annotated datasets that reflect the specific defects and conditions relevant to reinforced concrete bridges. Another issue is the selection and optimisation of detection models that can balance accuracy, speed, and computational efficiency, especially when deployed on mobile or edge devices. Finally, it is crucial to evaluate the coherence between AI-generated outputs and the classification criteria used in engineering codes and guidelines, such as the Italian Guidelines [2]. However, a major drawback of the existing AI-based detection methods is related to the limited range of considered defects, which makes them unusable in inspection frameworks that require full code-compliance.

Given the mentioned challenges, this study aims to address these challenges by proposing and validating a DL-based approach for defect detection in prestressed and reinforced concrete bridges using a YOLOv8 model. The research objectives are fourfold: (1) to define a defect taxonomy aligned with the Italian Guidelines and commonly encountered degradation patterns in the field; (2) to create a labelled image dataset collected from inspection campaigns in the Basilicata region; (3) to train and evaluate YOLOv8 object detection models using appropriate metrics such as precision and recall; and (4) to evaluate the trained model for assessing its potential contribution in supporting inspectors’ activities during bridge visual inspection, also using the developed Graphical User Interface (GUI). Indeed, the developed tool is intended to facilitate the evaluation workflow in code-mandatory routine inspections rather than replace human judgment and decision-making, whose context awareness cannot be (currently) simulated by algorithms. Moreover, human oversight remains necessary for accountability purposes in bridge management authorities’ procedures.

The structure of this paper is organised as follows. The remainder of this section reviews the state of the art in AI applications for civil infrastructure inspection, with a focus on bridge-related studies and performance metrics reported in the literature and the considered defect taxonomy. Section 2 presents the proposed methodology, including the inspection framework defined by the Italian Guidelines and the characteristics of the YOLOv8n model. In Section 3, the composition of the dataset and the training procedures are presented. Section 4 illustrates the application of the model to a real-world case study and discusses the results obtained. Section 5 discusses the implementation of multimodal attention mechanisms devoted to improving the detection performance of the YOLO model and presents the developed Graphical User Interface. Finally, Section 6 concludes the paper with final remarks and directions for future work.

### Related Work: Computer Vision Applications for Civil Infrastructure Inspection

Civil Engineering is among the fields benefiting from AI advancements, using it to enhance various aspects of infrastructure design, construction, and maintenance. Some key applications include:

- Predictive modelling to estimate the lifespan of structures.
- Optimised resource management.
- Automated inspection through computer vision technologies.
- Data analysis to forecast degradation and reduce maintenance costs.

Driven by digital transformation [9] and big data, interdisciplinary studies based on deep learning have gained increasing attention in the field of automated inspection. This section analyses several deep learning algorithms used in the inspection and maintenance of civil infrastructure. Roads, buildings, and bridges are fundamental components of both urban and rural networks, and their upkeep is essential to ensure safety, efficiency, and sustainability. For this reason, many researchers have investigated how deep learning algorithms can be leveraged to improve infrastructure maintenance processes.

For example, Ni et al. [10] focused on detecting and localising pavement damage on bridge decks using deep learning techniques. Specifically, five damage categories (longitudinal cracks, transverse cracks, alligator cracks, potholes, and repairs) were initially identified using YOLOv7. Then, a revised LaneNet model was employed to detect lane markings via semantic segmentation. In the final phase, a post-processing algorithm enabled the localisation of damage in relation to bridge lanes.

In the context of stability and damage control in the aftermath of a seismic event, Kizilay et al. [11] investigated the use of deep learning models for real-time earthquake damage assessment based on drone imagery. The study compares various architectures, including YOLOv8, Detectron2, and a modified VGG16, to evaluate their effectiveness in detecting damaged buildings. Models were tested for identifying whether a building had collapsed and for classifying damage levels (severe, moderate, minor, or none), as well as for detecting wall damage (vertical and horizontal cracks). The results show that YOLOv8 offers an optimal balance between accuracy and speed.

Zou et al. [12] proposed a method for multi-category damage detection and safety assessment of reinforced concrete structures after earthquakes. YOLOv4 was used to detect a few types of structural damage (e.g., fine and wide cracks, concrete spalling, and exposed or bent rebars), and was enhanced with depthwise separable convolutions. Finite element models were also employed to analyse damage progression and develop assessment criteria. Each structural component (beam, column, and wall) was evaluated based on damage type, location, and severity.

Dogan et al. [13] developed an algorithm to distinguish corrosion-induced structural damage from seismic damage in reinforced concrete buildings. A dataset built from post-earthquake inspections was used for training and testing. The algorithm correctly classified 21 out of 25 damage images observed after the 2020 Samos (Greece) earthquake [14], achieving a validation success rate of about 84%.

The accurate assessment of historic buildings, of their condition and signs of deterioration, is crucial to design respectful and effective restoration strategies. Mishra et al. [15] explored AI-assisted visual inspection of heritage buildings, comparing the performance of ChatGPT-4o and YOLOv5. The main goal was to evaluate the ability of these tools to identify and describe surface deterioration. ChatGPT used pre-trained models and the ICOMOS-ISCS glossary to describe types of degradation, while YOLOv5 was trained on a manually annotated dataset. ChatGPT proved effective in providing qualitative damage descriptions but lacked localisation accuracy, whereas YOLOv5 delivered more precise localisation, at the cost of requiring a dedicated training set.

Katsigiannis et al. [16] proposed a deep learning approach to detect cracks in brick masonry façades using transfer learning due to limited data. A pre-trained deep convolutional neural network was fine-tuned for crack detection. Four training strategies were evaluated, with the best performance achieved using end-to-end MobileNetV2 [17] and InceptionResNetV2 [18] with data augmentation and Xception [19] without augmentation.

Karimi et al. [20] developed a deep learning model to detect cracks in various materials found in cultural heritage (CH) structures. YOLOv5 performance was compared across images of stone, brick, clay, tiles, and concrete, using transfer learning with weights pre-trained on the COCO dataset.

Even modern concrete buildings can deteriorate over time due to various factors. Typical issues include carbonation-induced degradation, reinforcement corrosion, and structural cracking from high loads or ground settlement. Identifying such defects is critical for planning effective maintenance and enhancing durability and safety. Liu et al. [21] focused on identifying and quantifying damage in concrete structures. Their model calculates parameters like crack width, length, and angle, while crushed areas are measured by damaged surface area. Multi-view geometric reconstruction was used to create 3D models of the structures, aiding in more accurate assessments. However, the model was focused only on cracks.

As for automated bridge inspections, these studies have addressed the degradation of bridge bearings, whose failure can compromise load transmission and boundary conditions. Wang et al. [22] developed a method to detect and classify the condition of steel bridge bearings. Two models were proposed: BearDet for bearing detection in inspection images and BearCla for degradation classification. A multiscale training strategy was introduced to enhance detection accuracy by dynamically varying input image size. Feature Pyramid Networks (FPNs) were also employed to address multiscale challenges.

Numerous deep learning algorithms for detecting surface damage on infrastructure exist in the literature. The choice of model depends on its specific strengths. For instance, Huang et al. [23] developed BridgeNet, an architecture that integrates Mask R-CNN with the Swin Transformer and the CARAFE [24] upsampler to enhance global feature extraction. BridgeNet combines the long-range relational capabilities of Transformers with the segmentation precision of Mask R-CNN, effectively identifying damage types like cracks, spalling, corrosion, honeycombing, and blockage.

Some authors have compared Transformer-based models and CNNs for segmentation tasks. Fukuoka et al. [25] used semantic segmentation to detect delamination and exposed rebars. They compared SegFormer (a Transformer-based model) with SegNet (CNN-based), trained on datasets with varying image sizes. Performance was evaluated across three datasets: unprocessed, 224-pixel segments, and 448-pixel segments.

Yamane et al. [26] developed a framework combining Structure from Motion (SfM) and Visual Question Answering (VQA) to estimate bridge damage causes. SfM was used for 3D reconstruction and contextual image positioning, while a VQA model trained on bridge image datasets responded to structural condition queries. Features were extracted via Faster R-CNN (pre-trained on Visual Genome) and paired with text input to produce answers. Yes/No questions had 99.1% accuracy, component naming had 67.4%, and damage type recognition had 68.9%, with an overall accuracy of 81.2%.

Su et al. [27] proposed an enhanced YOLOv3-based damage detection approach for complex scenes, addressing background noise and scale variation.

Yu et al. [28] propose a modified one-stage object detection model called YOLOv4-FPM, which enhances YOLOv4 with focal loss to improve accuracy in complex backgrounds and pruning to reduce model size and accelerate detection, achieving real-time crack detection performance on bridge images.

Zhao et al. [29] propose an enhanced damage detection framework called YOLOv5s-HSC for concrete dams (primarily cracks and spalling phenomena), integrating Swin transformer blocks and coordinate attention modules to improve feature extraction in complex backgrounds and achieve higher detection accuracy. Additionally, detected damages are mapped onto a photogrammetric 3D reconstruction model for precise spatial localization.

Dong et al. [30] introduced YOLOv8-CD for detecting concrete cracks, using enhancements like LSKA (Large Separable Kernel Attention), Ghost, GSConv, and VoV-GSCSP. These modules improve feature extraction, reduce computational complexity, and enhance detection performance.

Ruggieri et al. [31] developed BRIDE-YOLO to detect seven classes of defects in reinforced concrete infrastructure. The approach uses an improved YOLO model with attention mechanisms to focus on relevant image areas. Eigen-CAM [32] was used to visually explain model decisions, and a GUI was developed to facilitate interaction with technicians.

YOLO can also be deployed on lightweight devices using optimised versions and suitable hardware accelerators. Zakaria et al. [33] implemented real-time deep learning-based bridge inspections on edge devices. YOLOv5s was used for detecting cracks and spalling, and an EfficientNet-b0-based UNet for defect sizing. Models were quantised for edge deployment. Augmented/mixed reality (AR/MR) interfaces enabled inspectors to validate and adjust AI outputs in real time. Chen et al. [34] presented a portable system for the real-time detection of bridge damage using drones and CD-YOLOv8. The algorithm was optimised for compact platforms like the Jetson Xavier NX.

It must be noted that, although some of the previous applications provided valuable advancements in concrete surface defect recognition, they focused on a very limited variety of classes, making them unsuitable for developing a tool able to help inspectors in interpreting routine inspection images to determine the structural risk scores, according to codes. For this reason, the main objective of this study is to develop and test a pipeline for damage detection working on a wider range of defects to obtain code-conforming evaluations.

## 2. Methods

### 2.1. Object Detection Algorithm and Evaluation Metrics

The methodology presented here is grounded in the Italian Guidelines for Bridge Risk Classification and Management (the current code for bridge management and assessment in Italy) and employs a deep learning approach trained on annotated datasets derived from inspections of existing bridges. The system focuses on detecting surface-level defects such as cracks, concrete spalling, rust stains, and water infiltration, providing a structured output aligned with standard defect sheets.

The VIADUCT procedure employs YOLOv8n as the core object detection model due to its optimal balance between detection accuracy, processing speed, and computational efficiency. YOLO, introduced by Redmon et al. [6], reframes object detection as a single-stage regression problem. The image is divided into an SxS grid, and each cell predicts bounding boxes and class probabilities. YOLOv8, released by Ultralytics in 2023, features improved architecture with an anchor-free detection head, better loss functions, and enhanced feature fusion techniques inspired by FPN and PAN. YOLOv8 is available in various model sizes; this study adopts YOLOv8n (Nano), suitable for limited computational resources.

In large-scale bridge inspection workflows—where thousands of images need to be analysed, often with limited access to high-performance hardware—YOLOv8n ensures fast and reliable inference even on low-power devices. Its lightweight architecture makes it particularly suitable for integration into mobile applications, allowing inspectors to perform real-time, on-site damage recognition directly from a smartphone or tablet. While more recent models, such as YOLOv9 or YOLOv12, introduce architectural innovations and may offer improved accuracy on benchmark datasets, their higher computational requirements limit their usability in field conditions [35]. Even though the pipeline developed herein is meant for desk workflows regarding previously collected on-site images, YOLOv8n also represents a robust, accessible, and scalable solution when portable tools are developed.

In order to evaluate the detection capability of the trained algorithms, one must consider that the Intersection over Union (IoU) metric is commonly used to assess the degree of overlap between predicted bounding boxes and the ground-truth bounding boxes. IoU is calculated as the ratio of the intersection area to the union area of the two boxes (ranging from 0.0 to 1.0), and it is critical for determining whether a predicted box is accurate. A detection is a true positive (TP) when IoU is above a certain threshold (say, 0.5), and the detected class is correct. Based on this, the metrics used were Recall (R), Precision (P), and F1-Score defined as follows:

(1) $$R=\frac{TP}{TP+FN}$$

(2) $$P=\frac{TP}{TP+FP}$$

(3) $$F1\;score=2\cdot \frac{P\cdot R}{P+R}$$

where

- TP: True positive
- FP: False positive
- TN: True negative
- FN: False negative

**Figure 1:**
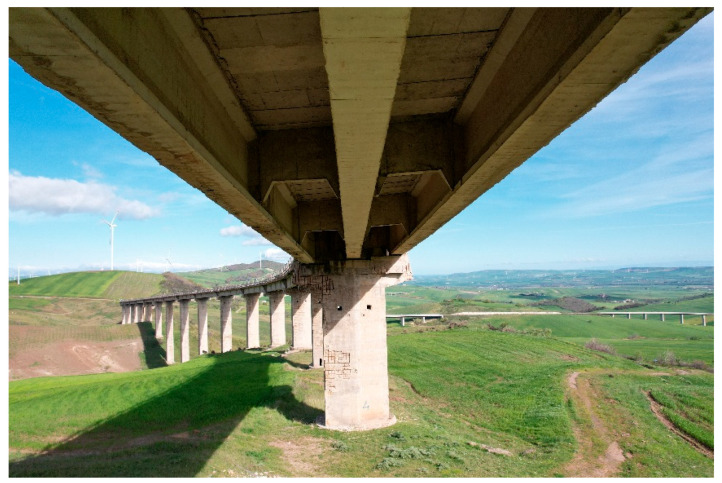
Typical panoramic inspection image.

More details about the specific meaning of *P*, *R* and *F*1 *score* will be given at a later point in the text. In this study, classification (detection of the right defects) is more important than the precise localization of defects. The objective is to identify the correct classes of damage associated with each image, with a minor focus on the exact position of the bounding boxes. Therefore, IoU is less relevant in this context and for this reason was set to a minimum value of 0.1. An additional reason that makes the application of the above-mentioned metrics with standard IoU values (e.g., 0.5) unfeasible is that during inspections, inspectors often take panoramic images that include several external elements with a large portion of the background possibly made of vegetation or the landscape, with defects that occupy a very small portion of the image (Figure 1). Due to this, the detection of the exact location of surface damage in bridge elements is more difficult with respect to standard, zoomed and orthogonal images.

### 2.2. Code-Conforming Approach for Surface Damage Detection

The assessment of existing bridges in Italy is governed by the Guidelines for the Classification and Management of Risk, Safety Assessment, and Monitoring of Existing Bridges, issued in 2020 by the Italian Ministry of Infrastructure and Transport [2]. These guidelines introduce a multi-level and multi-risk approach to infrastructure safety, recognising the complexity and interdependence of various risk components in the management of ageing civil structures.

The multi-level framework consists of six progressive levels, from Level 0 to Level 5:

- Level 0: *Census*—gathering basic administrative and structural data on each bridge.
- Level 1: *Visual Inspection*—aimed at documenting observable defects through on-site surveys and photographic documentation.
- Level 2: *Preliminary Risk Classification*—integrates data from Levels 0 and 1 to provide a synthetic risk index for prioritisation named Class of Attention (CdA).
- Levels 3–5: *In-Depth Evaluations*—these include detailed structural analysis (e.g., finite element modelling), load testing, structural health monitoring, and full safety assessments, which are required for bridges flagged as potentially critical at Level 2.

This hierarchical structure ensures that resources and in-depth analyses are focused on the structures most in need, while all bridges remain under systematic surveillance through the initial levels.

In parallel, the guidelines adopt a multi-risk model, accounting for:

- Structural and Foundational Risk;
- Seismic Risk;
- Hydraulic Risk;
- Landslide (Geological) Risk.

Among these, the Guidelines consider the structural and foundational risk to be the dominant component in determining the overall risk index [3]. Within this domain, the vulnerability of the structure, defined as its inherent predisposition to deteriorate or fail under service or hazard conditions, plays a pivotal role. In turn, the vulnerability is strictly linked to the defects detected through on-site inspections on the structure, since they can affect the operation and safety.

**Figure 2:**
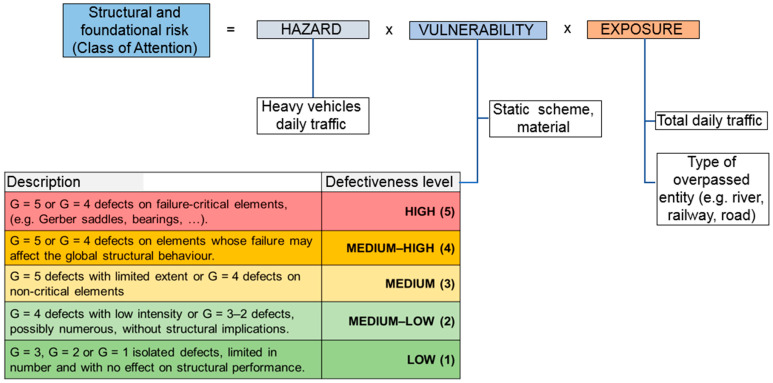
General framework for structural and foundation risk assessment according to the current Italian code.

A summary of the structural and foundation risk evaluation process is reported in Figure 2, where its evaluation (Class of Attention) is the product of Hazard, Vulnerability, and Exposure, in accordance with a probabilistic risk-based approach.

Within this formulation, Vulnerability (whose relative influence is much higher than Hazard and Exposure as reported in [3]) is directly governed by the defectiveness level, which is defined on the basis of the severity (G) of the detected defects and their location within the structural system.

In particular, defects with high severity levels (G = 5 or G = 4), occurring on failure-critical elements or on elements whose failure may affect the global structural behaviour, lead to high or medium–high defectiveness levels, which dominate the vulnerability term. As a consequence, even in the presence of moderate hazard or exposure conditions, the overall risk may rapidly increase. This highlights how the risk assessment is founded upon the detection of the worst severity defects across the structure.

To enhance standardisation and reproducibility of the Level 1 inspections, the Guidelines provide 20 distinct inspection forms, each tailored to specific bridge components and materials. Among these, six forms are dedicated to reinforced concrete (RC) and prestressed reinforced concrete (PRC) elements, including:

- RC abutments (form no. 1);
- RC piers (form no. 3);
- RC columns (form no. 8);
- RC girders and transverse beams (form no. 14);
- PRC girders and transverse beams (form no. 15);
- RC slabs (form no. 18).

Each form includes a predefined list of potential defect typologies, along with a classification system for their severity (G), extent (K1), and intensity (K2). The severity index G ranges from 1 to 5 (min-max), which identifies the possible impact of a specific defect on the bridge’s structural vulnerability. It is worth noting that extent indicates the spatial distribution of the defect, i.e., how much of the element surface is affected (e.g., localised vs. widespread damage), while intensity refers to the criticality of the defect at the affected area, i.e., how serious the damage is in terms of depth, deterioration level, or impact on functionality. When a defect is detected, both extent and severity can assume specific values (0.2, 0.5 or 1.0). This combination of parameters allows for assigning an overall level of defectiveness to the element at hand. Lastly, the worst level of defectiveness among all the structural components would be assigned to the entire bridge and would affect the overall structural risk evaluation.

Therefore, in order to develop an AI-based defect detection pipeline that is fully compliant with the national regulatory framework and can help inspectors during their field and subsequent damage detection work, it is necessary to ground the defect detection procedure in the typological system codified by the Guidelines.

To this end, the six RC-related inspection forms were carefully reviewed, and a unified typological list of defects was compiled (Table 1). This unified list consolidates the defect entries from all six forms, eliminating redundancies where identical or semantically equivalent defects appear across multiple forms. The result is a streamlined and exhaustive set of unique defect classes applicable to RC and PRC elements, which preserves full alignment with the official inspection procedures. Table 1 shows the original defect code, its description and the original form it belongs to, along with its severity (G).

This typological scheme served as the foundation for annotating the image dataset used in the training phase of the YOLOv8 deep learning model. Each annotated instance corresponds to one of the standard defect types, as defined in the unified list of Table 1, thereby ensuring that the model learns to detect only those defects that are recognised and actionable within the current regulatory system.

**Figure 3:**
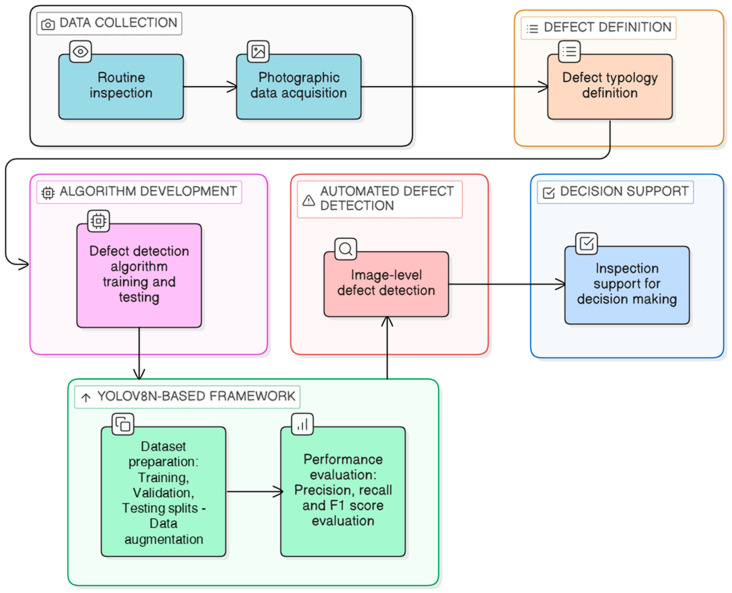
Framework for AI-assisted visual inspection.

In this way, the proposed AI-based methodology does not attempt to replace or circumvent existing standards but rather enhances their implementation by providing inspectors with a supportive tool capable of automatically localising and classifying visible damage, while reducing subjectivity and facilitating large-scale analyses of bridge conditions (Figure 3).

For transparency purposes, during the writing of this paper, generative artificial intelligence tools were employed solely for the translation into English and for grammatical and stylistic revisions of the text, without influencing data analysis or scientific outcomes.

### 2.3. Declaration of Generative AI in Scientific Writing

During the preparation of this manuscript/study, the authors used ChatGPT 5.2 for the purposes of translation from Italian and English editing. The authors have reviewed and edited the output and take full responsibility for the content of this publication.

## 3. Model Training

### 3.1. Bridge Dataset

**Figure 4:**
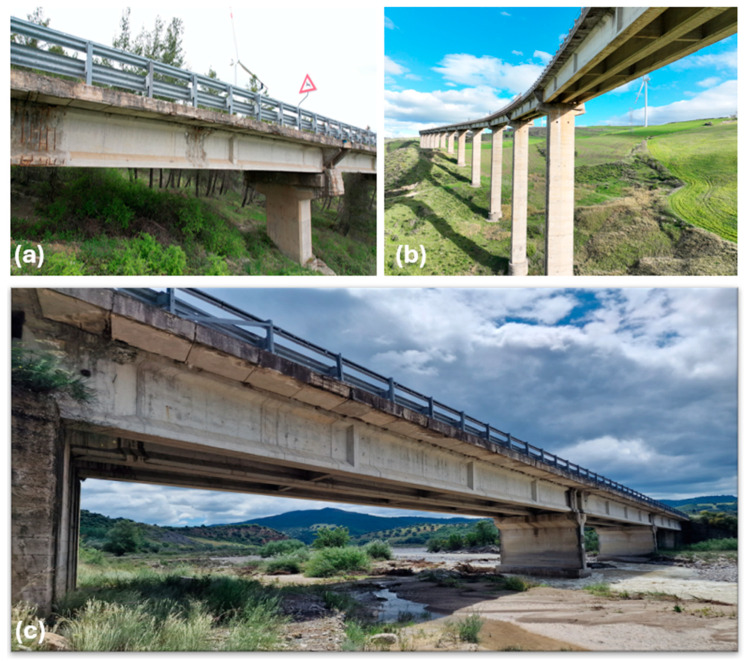
Views of some of the examined bridges: (**a**) bridge#1, (**b**) bridge #7 and (**c**) bridge #8.

Eight bridges located in the Basilicata region were selected, all with girder-type superstructures made of prestressed concrete (PC) with cast-in-place or precast components (Figure 4). Most bridges have rectangular piers and deep foundations. Detailed data on the geometry, span count, and girder configuration are summarised in Table 2. Some bridges feature multiple spans and variable girder spacing; for example, Bridge No. 3 shows changing girder numbers across spans. Due to an NDA agreement with the managing authority, the exact name and location of the bridges cannot be declared.

A total of 566 photographs were collected and annotated across the eight bridges during the routine inspections and used in this study (Table 2).

### 3.2. Image Annotation and Augmentation

Annotations were performed using Roboflow [36], following the supervised learning paradigm. Defects were labelled according to the official nomenclature in the Guidelines (e.g., Table 1), mapped to specific structural components using Inspection Sheets No. 1, 3, 8, 14, 15, and 18 as mentioned before. The dataset was split into training, validation, and test subsets. The training set was used to optimise model weights, the validation set to monitor performance, and the test set to assess generalisation.

It is important to underline that data augmentation was used to enhance the database’s significance and improve the training features. Data augmentation techniques were Gaussian blur (0–2.5 px) and exposure variation (±15%), which simulated poor image sharpness and lighting variability, respectively. This made it possible to increase the dataset size to 1045 images. Data augmentation only affected the training subset, while other subsets remained unchanged, as shown in Table 3.

Table 4 lists the full mapping between defect classes (Class Id), their descriptions (Class name), severity grades (G) according to the Italian code [2] and the total number of annotations.

Among the 46 possible defects, a total of 11 classes were found in the available images, including general and specific damage types. Some defect labels (e.g., “Crack”) were generalised due to the model’s inability to distinguish among structural elements and among vertical, transverse or horizontal crack patterns.

**Figure 5:**
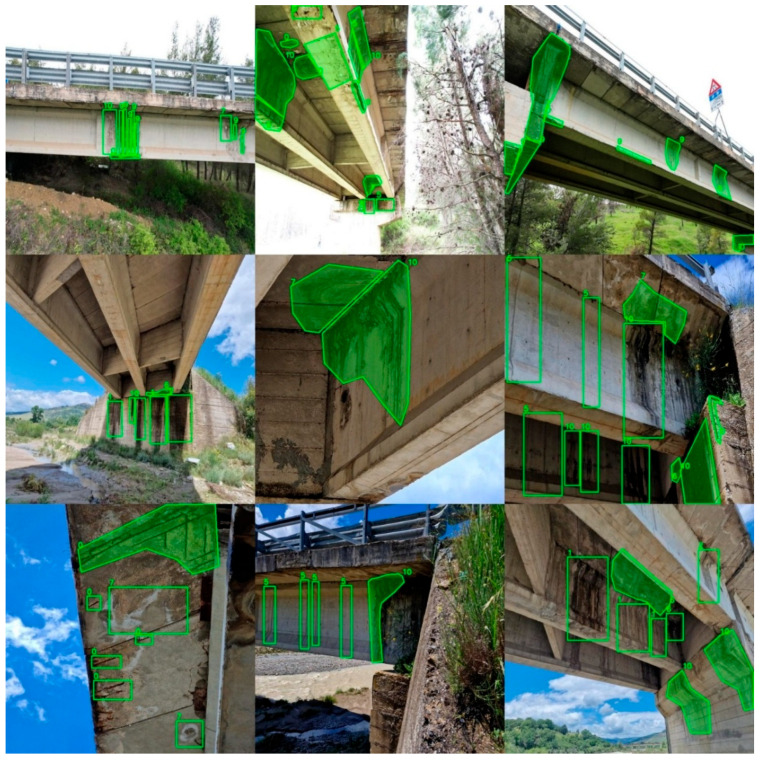
Example of image annotations through polygons and rectangles (pictures belonging to the training set). The numbers correspond to the classes in Table 4.

Two annotation types were adopted: bounding boxes and polylines, with careful attention to excluding irrelevant background and avoiding noise. Annotation quality was prioritised to prevent overfitting and ensure model robustness (Figure 5).

It must be noted that, during annotation, two identical classes were defined (i.e., class 8 and class 9 were both defined as Exposed/corroded stirrups). Therefore, in the performance evaluation phase, they were joined, and class 9 is no longer shown. Moreover, the limited number of annotated instances for the “Protruding anchor bars” class represents a learning limitation. However, this class was retained due to its high structural relevance in bridge safety assessment, despite its low occurrence in the available dataset.

The YOLOv8n model was trained using Python 3.13.5 (Ultralytics YOLO library), with default parameters except for an input image size of 640 × 640 and 50 training epochs. This configuration provided a good balance between computational efficiency and detection accuracy.

## 4. Model Performance Evaluation

As previously stated, the comparison between the YOLO predictions and the benchmark represented by the annotated images used as test set is performed at the defect level. For each class, True Positive (TP), False Positive (FP) and False Negative (FN) defects were evaluated to compute Precision, Recall and F1 score, neglecting the IoU value and focusing only on the presence of defects.

In this context, Precision (P), computed through expression (1), measures the ability of the algorithm to avoid false positives (higher precision means fewer FPs), which are defects absent in the inspector’s evaluation (and in the annotation). Recall (R), which is computed by expression (2), quantifies the ability to correctly identify existing defects, avoiding or reducing false negatives (fewer FNs); the F1 score provides a single, balanced measure of accuracy and sensitivity (expression 3). All these performance metrics are bounded between 0.0 and 1.0, where values closer to 1.0 indicate better model performance. It is worth noting that, in the context of structural health monitoring, false negatives are more critical than false positives, since it is important not to miss defects that are actually affecting the structural elements.

Table 5 reports the results in terms of detections across the 44 pictures in the test dataset. Beyond the Class Id (defect identification) and its severity, the ground truth true positives (GT_TP) are reported as the number of times that a defect is found across the images. The last six columns show Precision, Recall and F1 score values for each specific Class Id.

**Figure 6:**
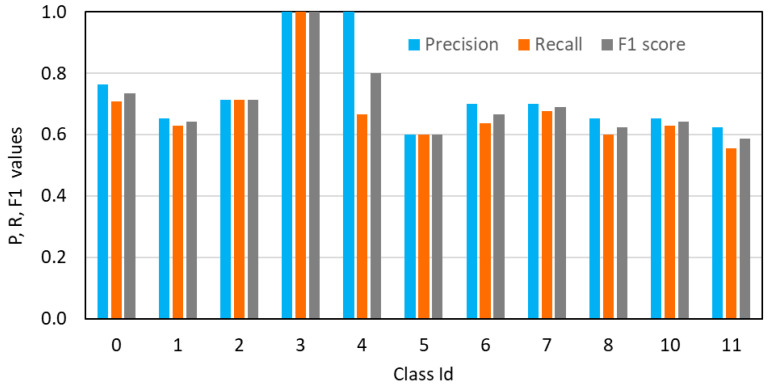
Precision, Recall and F1 score values over the classes.

The class-wise performance metrics reported in Table 5 (and Figure 6) should be interpreted with caution due to the limited size and imbalance of the test dataset. In particular, classes 2, 3 and 4 are represented by only 7, 2 and 2 ground-truth instances, respectively, which restricts the statistical reliability of the corresponding precision and recall values and prevents any strong generalisation.

Despite these limitations, the overall results can be considered acceptable given the challenging classification task, which involves 11 defect categories learned from a relatively small dataset. Lower performance is observed for classes associated with lower severity levels (e.g., classes 5 and 10, severity G = 2), whose identification is mainly relevant for maintenance prioritisation rather than for immediate safety assessment.

Conversely, defects characterised by higher severity levels exhibit more stable and satisfactory performance. This aspect is particularly relevant within the adopted risk assessment framework, where defect severity directly governs the vulnerability component of the Hazard–Vulnerability–Exposure formulation (Figure 2). As a consequence, the correct identification of high-severity defects (G = 5 and G = 4) plays a dominant role in the estimation of structural vulnerability and, ultimately, in the assignment of the Class of Attention.

Since previous metrics are related to a low and unusual IoU threshold value, to ensure a fair comparison with state-of-the-art methods, using standard metrics, we also computed the mAP@0.5 = 0.482, which is lower than in other studies. For instance, in Refs. [26,30] mAP@0.5 is equal to 0.798 and 0.594, respectively, which are significantly better, although the algorithms were trained to detect only a few defects like spalling and exposed bars instead of providing a code-compliant framework.

**Figure 7:**
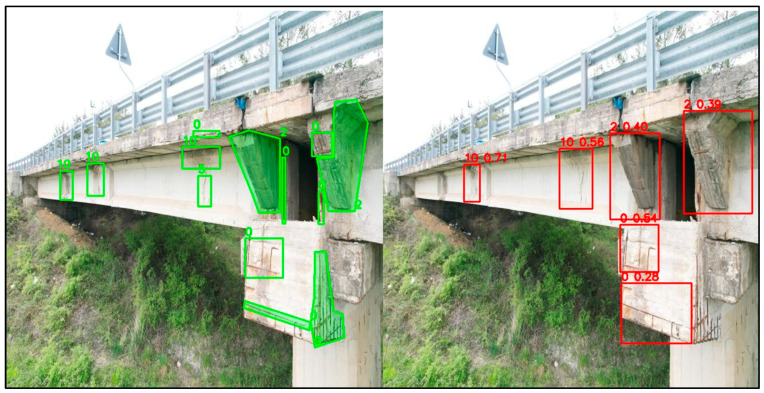
GT and YOLO prediction comparison on bridge #1. The numbers correspond to classes in Table 4.

In the following, some detection capability examples are reported. Figure 7 compares the ground truth annotations, shown in green on the left, with the model predictions, shown in red on the right, for a representative bridge inspection image. The ground truth, drawn with polygonal shapes, captures complex and elongated defects that extend over large portions of the structural elements, whereas the model outputs rectangular bounding boxes with associated confidence scores. Although the localisation is not always accurate (the problem related to wide-angle images), the model correctly identifies the presence and the type of most relevant defects in the pier and deck region. In this example, the only defect category that is not detected is class 5 (cracks near stirrups), which appears in the ground truth as a small and visually subtle feature within a highly textured area. Overall, the figure supports an image-level evaluation strategy, where the main objective is the correct identification of defect classes within an image rather than precise geometric localisation, which is consistent with infrastructure risk assessment practice, since the global condition is driven by the presence of the most severe defects rather than their exact spatial extent.

**Figure 8:**
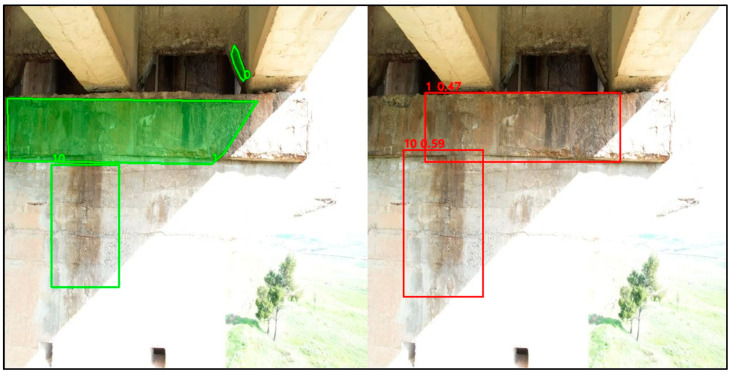
GT and YOLO prediction comparison on an image from bridge #7.

Figure 8 compares ground truth annotations, shown in green, and model predictions, shown in red. The ground truth highlights three different defect classes within the same image. A large polygon identifies class 1, washed-out or deteriorated concrete, along the cap beam supporting the deck; a vertical region on the pier is annotated as class 10, drainage streaks; and a small, localised polygon near the transverse beam corresponds to class 0, corroded or oxidised reinforcement. The model correctly detects class 1, washed-out or deteriorated concrete, and class 10, drainage streaks, producing bounding boxes that overlap the same structural regions. Conversely, the small-scale defect associated with class 0, corroded or oxidised reinforcement, is not detected, likely due to its limited spatial extent and visual subtlety within a complex background. Overall, the example confirms that the model reliably identifies dominant deterioration mechanisms at the image level, while very localised defects affecting reinforcement remain more challenging.

**Figure 9:**
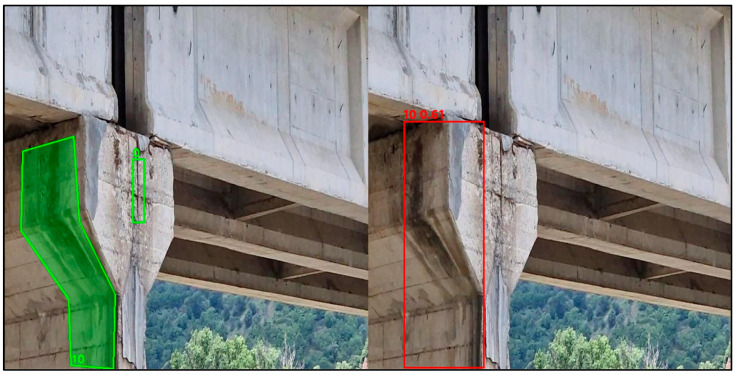
GT and YOLO prediction comparison on an image from bridge #8.

Yet, in Figure 9, the ground truth identifies an extended deterioration phenomenon along the pier surface, annotated as class 10, drainage streaks, together with a small, localised defect near the beam–pier interface corresponding to class 0, corroded or oxidised reinforcement. The model correctly detects the dominant deterioration mechanism, predicting drainage streaks (class 10) with a high confidence score and covering the same structural region affected by moisture-related staining. Conversely, the small-scale defect associated with corroded or oxidised reinforcement (class 0) is not detected, confirming the reduced sensitivity of the model to highly localised defects when embedded in complex textures, partially shadowed areas and non-orthogonal views. This example further highlights that the model is effective in identifying prevailing deterioration mechanisms at the image level, while very localised reinforcement-related defects remain more challenging to capture reliably.

## 5. Implementation of Multimodal Attention Mechanisms and Graphical User Interface

This section presents a comprehensive Graphical User Interface (GUI) system for automated bridge damage detection, integrating multiple computer vision techniques, including attention mechanisms, object detection, and video tracking. The system employs a tile-based processing architecture with advanced segmentation methods and real-time defect tracking capabilities.

### 5.1. System Architecture

The VIADUCT system v3.0 implements a PyQt5-based graphical interface that orchestrates multiple deep learning models for bridge infrastructure inspection. The interface is designed with a maximised full-screen layout, featuring a left control panel and a right visualisation area organised in tabbed views.

#### 5.1.1. Attention Mechanisms for Damage Detection in Images: SAM and U-Net Segmentation and Tile-Based Processing

The system integrates Meta’s Segment Anything Model (SAM) as a primary attention mechanism for image segmentation. SAM [37] provides interactive segmentation capabilities through point-based user guidance. Users can select foreground points (left-click) to include regions of interest or background points (right-click) to exclude unwanted areas. This interactive approach allows for the precise refinement of segmentation masks, enabling users to iteratively improve segmentation quality by providing spatial cues directly on the image.

SAM operates on the full image resolution and generates high-quality segmentation masks through its transformer-based architecture. However, this computational complexity results in slower processing times compared to the U-Net alternative, making it more suitable for scenarios requiring high precision and user-guided refinement.

The system employs a custom U-Net [38] architecture as an alternative attention mechanism, specifically designed for bridge damage segmentation. The U-Net model was trained on a dataset of 205 bridge images that were manually segmented using a dedicated annotation interface. This annotation tool, based on SAM, automatically saves segmentation masks and corresponding images in separate directories, ensuring proper data organisation for training.

The U-Net architecture consists of an encoder–decoder structure with skip connections, featuring double convolutional blocks with batch normalisation and ReLU activations. The encoder progressively downsamples the input through four levels (64, 128, 256, and 512 channels), followed by a bottleneck layer (1024 channels), and a symmetric decoder that reconstructs the segmentation mask at full resolution.

The key advantages of U-Net over SAM are:

- Speed: U-Net demonstrates significantly faster inference times, making it more suitable for real-time applications and batch processing scenarios.
- Deterministic output: Unlike SAM, U-Net does not support interactive point selection, providing consistent, automated segmentation without user intervention.
- Domain-specific training: The model is specifically trained on bridge infrastructure images, potentially offering better generalisation for this application domain.

A critical innovation in the system is the use of Gaussian blur for visualising non-segmented regions, rather than traditional black masking. When applying segmentation masks to input images, regions outside the mask (background or non-relevant areas) are processed using a Gaussian blur filter with a kernel size of 51 × 51 pixels and a standard deviation (σ) of 0, which defaults to automatic calculation based on kernel size. This approach creates a strong blurring effect that effectively de-emphasises non-relevant regions while maintaining visual context.

The blurred regions are blended with the original image using the segmentation mask as an alpha channel, where mask values of 1 preserve the original image and values of 0 apply the blurred version. This blur-based visualisation has been empirically demonstrated to perform better than black masking for several reasons:

- Context preservation: Blurred regions maintain spatial and colour context, allowing users to understand the relationship between segmented and non-segmented areas.
- Visual continuity: The gradual transition between focused and blurred regions reduces visual discontinuity compared to sharp black boundaries.
- Attention guidance: The blur effect naturally guides visual attention toward the sharp, segmented regions without creating harsh visual artefacts.
- Reduced cognitive load: Users can still perceive the overall scene structure, facilitating better understanding of the segmentation results.

To handle high-resolution bridge images and improve detection accuracy, the system implements a tile-based processing pipeline [39]. Each input image is divided into four non-overlapping tiles arranged in a 2 × 2 grid. The tiling algorithm calculates tile dimensions as half the image height and width, ensuring equal distribution of image content across tiles.

For each tile, the segmentation mask (from either SAM or U-Net) is applied, and the blur mechanism is used to de-emphasise non-segmented regions. YOLO object detection is then performed independently on each processed tile, allowing the model to focus on smaller, more manageable image regions.

After processing all four tiles, the system recomposes the original image by concatenating the processed tiles in their original spatial arrangement. The recomposition algorithm handles potential size variations between tiles by calculating maximum dimensions for each row and column, ensuring proper alignment. The final recomposed image displays all detected defects with their bounding boxes and confidence scores, providing a comprehensive view of the entire bridge structure.

The system implements Non-Maximum Suppression (NMS) to eliminate duplicate detections that may occur at tile boundaries or due to overlapping bounding boxes. The NMS algorithm uses OpenCV’s cv2.dnn.NMSBoxes function, which applies the standard NMS algorithm based on Intersection over Union (IoU) thresholding.

A critical feature of the interface is the user-adjustable IoU threshold control, allowing real-time modification of the NMS sensitivity. The IoU threshold slider ranges from 0.0 to 1.0 (default: 0.3), enabling users to balance between detection recall and precision. Lower IoU thresholds result in more aggressive suppression (fewer duplicates but potentially missing valid detections), while higher thresholds allow more detections to pass through (better recall but potentially more duplicates).

When the IoU threshold is modified, the system automatically re-runs the inference pipeline, ensuring that detection results are immediately updated with the new NMS parameters. This interactive control mechanism provides users with fine-grained control over the detection quality, allowing optimisation for specific bridge inspection scenarios.

#### 5.1.2. Video-Based Defect Tracking

For video analysis, the system implements a sophisticated homography-based tracking mechanism (HomographyTracker) designed to handle camera movement and perspective changes common in bridge inspection videos. The tracker compensates for camera motion by computing homography transformations between consecutive frames, stabilising the coordinate system for consistent defect tracking.

The tracking algorithm maintains a set of active tracks, each representing a detected defect across multiple frames. When new detections arrive, the system engages in the following:

- Transforms detection coordinates using the computed homography to account for camera movement;
- Calculates the IoU between transformed detections and existing tracks;
- Associates detections with tracks based on the IoU threshold (default: 0.3) and spatial distance;
- Updates track positions and maintains hit counters for each tracked object.

The tracker implements a minimum hit threshold (default: 5 frames) before a defect is counted, ensuring that transient detections or false positives are filtered out. Tracks that exceed a maximum age (default: 30 frames) without updates are removed, preventing the accumulation of stale tracks.

The system maintains class-specific counters that aggregate defect counts across the entire video sequence. These counters are updated in real-time and displayed in a dedicated sidebar panel adjacent to the video player, showing the format “N x DefectName” where N represents the total count of each defect type detected throughout the video.

The video analysis interface employs a horizontal split layout with the video player occupying 75% of the screen width and the defect summary list occupying 25%. This layout ensures that defect tracking information is immediately visible alongside the video playback, enabling real-time monitoring of detection progress.

The interface provides comprehensive control over all processing parameters:

- Confidence threshold: Adjustable from 0.0 to 1.0 (default: 0.25) for YOLO detection sensitivity;
- IoU threshold: Adjustable from 0.0 to 1.0 (default: 0.3) for NMS strength;
- Segmentation method: Dropdown selection between SAM, U-Net (augmented), U-Net (standard), or YOLO-only processing;
- Video playback speed: Adjustable playback rate for video analysis.

Upon the completion of image segmentation and inference, the system automatically navigates to the “Recomposed Image” tab, displaying the final results with all detected defects. Similarly, when processing videos, the interface automatically switches to the “Video” tab, ensuring that users are immediately presented with the most relevant visualisation.

Both image and video processing modes include dedicated defect summary panels. The image recomposition view displays a sidebar (25% width) listing all detected defects in the format “N x DefectName”, providing an immediate quantitative assessment of the bridge’s condition. The video view includes an identical summary panel that updates in real-time as the video is processed, showing cumulative defect counts.

The tile-based architecture provides several performance benefits:

- Parallel processing potential: Each tile can theoretically be processed independently, enabling future GPU parallelization;
- Memory efficiency: Processing smaller tiles reduces memory requirements compared to full-resolution processing;
- Detection accuracy: Smaller input regions allow YOLO to focus on local features, potentially improving the detection of small defects.

The choice between SAM and U-Net represents a trade-off between segmentation quality and processing speed. SAM offers superior interactive refinement capabilities but at the cost of computational time, while U-Net provides rapid, automated segmentation suitable for batch processing and real-time applications.

In order to measure the models’ performance, the recomposed image tab’s title is complemented with inference times for both the segmentation and object detection for each model’s inference. Moreover, the total processing time is also displayed.

Table 6 compares the computational performance of the three investigated approaches averaged across three sets of five images with increasing resolutions. The U-Net segmentation model exhibits nearly constant inference time, remaining below 0.4 s even when processing high-resolution images up to 19.96 Mpx, thus demonstrating strong scalability and suitability for large-scale inspection scenarios. In contrast, SAM shows a substantial increase in computational cost, with inference times exceeding 5 s for images above 9 Mpx, highlighting its limited efficiency for high-resolution structural imagery without further optimisation. Moreover, this segmentation method, in some cases, may need some refinement from the user in order to create a mask that effectively covers the bridge surface.

YOLO inference time increases approximately linearly with image resolution, ranging from 0.30 s at 0.41 Mpx to 1.22 s at 19.96 Mpx, reflecting the expected behaviour of detection-based architectures operating on larger spatial inputs. However, one must consider that the inference is performed separately on four images (the four tiles).

In summary, the results indicate that U-Net provides the best trade-off between segmentation accuracy and computational efficiency for high-resolution bridge inspection imagery, while SAM is considerably more resource-intensive, and YOLO offers intermediate performance with resolution-dependent latency.

All experiments were conducted on a laptop HP Zbook equipped with an Intel^®^ Core™ Ultra 7 155H processor (base frequency 1.40 GHz), 32 GB of RAM operating at 5600 MT/s, and a graphics unit with 4 GB of dedicated memory. The system runs a 64-bit operating system on an x64-based architecture, with approximately 1 TB of available storage. All deep learning models, including YOLO for object detection, the Segment Anything Model (SAM) for interactive segmentation, and the U-Net architecture for automated segmentation, were executed in a CPU-only environment, without GPU acceleration. This configuration was intentionally maintained to assess the computational feasibility of the proposed framework under resource-constrained conditions typical of real-world inspection scenarios.

**Figure 10:**
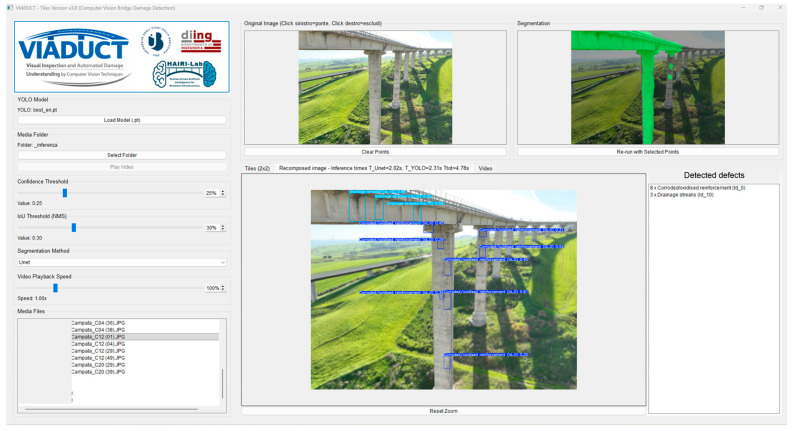
VIADUCT Graphical User Interface.

The VIADUCT interface (Figure 10) represents a comprehensive support tool for bridge damage detection, integrating multiple state-of-the-art computer vision techniques within an intuitive graphical framework. The system’s attention mechanisms, tile-based processing, blur visualisation, and video tracking capabilities provide inspectors with useful tools for infrastructure assessment, while the interactive controls enable fine-tuning for specific inspection scenarios. Moreover, as more advanced YOLO object detection models become available, they can be readily adopted by simply selecting them via the “Load model” button. It is worth noting that the media file box located in the bottom-right corner has been obscured to protect potentially sensitive information (bridge names). The tool is intended to allow for a desk operational workflow able to analyse previously collected bridge inspection images, possibly grouped into span folders. Then, the worst defect(s) in each span can be detected, and the overall defectiveness level can be assessed.

For demonstration purposes, a video is publicly available on the internet [40].

## 6. Conclusions

This paper presented a computer vision framework to support routine bridge visual inspections. Unlike approaches aimed at detecting a limited number of major defects, the proposed framework is designed to recognise a broad set of defect classes consistent with those required by standard inspection forms, with the objective of assisting inspectors in managing the large volume of visual information collected during routine surveys.

The damage detection module is based on the YOLOv8n algorithm, whose detection capabilities are discussed through representative examples that show the model is generally capable of identifying the prevailing deterioration mechanisms affecting structural elements, even when operating on wide-angle panoramic images characterised by complex backgrounds, variable lighting conditions, partial visibility of multiple components, and non-orthogonal viewpoints. In this context, extended and visually dominant defects are more reliably detected, while small and highly localised phenomena, such as limited areas of corroded reinforcement embedded in textured or shadowed regions, remain more challenging. From a methodological standpoint, the detection capability of the YOLO model was evaluated, attributing a lower importance to the precise localisation (intersection-over-union metrics) in order to privilege the image-level defect presence classification task as the primary objective. This choice is motivated by the nature of real-world inspection images, which are panoramic-like and often include large amounts of background.

The disadvantages brought by the type of inspection images suggested the implementation of multimodal attention mechanisms, such as semantic segmentation of the bridge surface using SAM and U-Net approaches, which permitted to adopt a guided or a deterministic segmentation, respectively, with different inference speeds. Moreover, tile reduction was developed to improve the YOLO inference results, activating the attention on limited portions of the image. These attention mechanisms were included in the Graphical User Interface, which permitted full control of the VIADUCT damage detection pipeline. The attention mechanisms are also adopted to partially overcome the dataset imbalance and limited sample size used in the YOLO model training. However, given how the GUI is designed, it will permit the replacement of the object detection model as soon as a new and more effective model becomes available.

Lastly, the entire VIADUCT pipeline is intended as a simple and friendly tool to support, rather than replace, inspectors in processing huge amounts of data. From this perspective, the entire process was conceived and will be further developed in accordance with the Human-In-The-Loop principles, which aim to actively include humans in the evaluation processes to give full accountability and traceability features to the whole infrastructure management process.

## Figures and Tables

**Table 1:** List of defects in RC and PRC elements.

Code	Defect Description	Forms	Severity G
c.a-c.a.p._1	Passive moisture stains	1, 3, 8, 14, 15, 18	1
c.a-c.a.p._2	Active moisture stains	1, 3, 8, 14, 15, 18	3
Dif_Gen_1	Drainage streaks	1, 3, 8, 14, 15, 18	3
c.a-c.a.p._3	Washed-out/deteriorated concrete	1, 3, 8, 14, 15, 18	3
Dif_Gen_2	Water ponding	1, 3, 14, 15	2
c.a-c.a.p._4	Honeycombs	1, 3, 8, 14, 15, 18	2
c.a-c.a.p._5	Spalling of concrete cover	1, 3, 8, 14, 15, 18	2
c.a-c.a.p._6	Corroded/oxidised reinforcement	1, 3, 8, 14, 15, 18	5
c.a-c.a.p._7	Minor crazing cracks	1, 3, 8, 14, 15, 18	1
c.a-c.a.p._8	Horizontal cracks	1, 3, 8	2
c.a-c.a.p._9	Vertical cracks	1, 3, 8	2
c.a-c.a.p._10	Diagonal cracks	1, 3, 8, 14, 15, 18	5
c.a-c.a.p._11	Cracks at column joint	1, 3, 8	3
c.a-c.a.p._12	Deteriorated construction joints	1, 3, 8, 14, 15, 18	1
Dif_Gen._3	Impact damage	1, 3, 8, 14, 15	4
Dif_Gen._6	Out-of-plumb	1, 3, 8	5
Ril-Fond_1	Scour	1, 3	5
Ril-Fond._2	Embankment washout	1	1
Ril-Fond._3	Embankment distress—deformations	1	2
Rif-Fond._4	Embankment distress—stability	1	4
Rif-Fond._5	Foundation movements	1, 3	5
c.a-c.a.p._13	Compression cracks	1, 3, 8	4
Dif_Gen._4	Characteristic cracks at bearing areas	1, 3	3
c.a-c.a.p._15	Cracks near stirrups	3, 14	2
c.a-c.a.p._16	Exposed/corroded stirrups	3, 8, 14, 15	3
c.a-c.a.p._23	Broken stirrups	3, 8, 14	4
c.a-c.a.p._17	Deformed longitudinal reinforcement	3, 8, 14, 15	5
c.a-c.a.p._18	Longitudinal cracks	14, 18	2
c.a-c.a.p._19	Transverse cracks	14, 15, 18	5
c.a-c.a.p._21	Washed-out/deteriorated concrete at ends	14, 15	4
c.a-c.a.p._22	Cracks/separation in transverse beams	14, 15	3
c.a-c.a.p._24	Defects in Gerber saddles	14, 15	5
Dif_Gen._5	Water ponding in box girders	14, 15	5
c.a.p._1	Capillary cracks at anchorages	15	1
c.a.p._2	Unsealed anchor head ends	15	2
c.a.p._3	Detachment of end blocks	15	1
c.a.p._4	Cracks on web along cables	15	2
c.a.p._5	Cracks along bulb flange	15	2
c.a.p._6	Exposed sheaths	15	2
c.a.p._7	Degraded sheaths and corroded wires	15	4
c.a.p._8	Visible corroded bonded wires	15	4
c.a.-c.a.p._25	Cracks at beam–slab interface	18	2
c.a.p._9	Reduction in prestressing reinforcement	15	5
c.a.p._10	Internal moisture	15	2
c.a.p._11	Exposed/corroded reinforcement at ends	15	2
c.a.p._12	Protruding anchor bars	15	5

**Table 2:** Characteristics of Bridge in the dataset.

Bridge	Total Length [m]	Avg. Span Length [m]	No. of PRC Spans	No. of Deck Beams	No. of Images
1	163	33	5	3	36
2	123	31	3	4	30
3	260	29	9	3	40
4	230	33	7	3	22
5	413	52	5	4	58
6	140	33	4	3	15
7	966	42	23	3	285
8	102	34	3	3	80
				Total	566

**Table 3:** Dataset before and after augmentation.

Subset	Images (Before Augmentation)	Images (After Augmentation)	Variation	Percentage of Total (After Augmentation)
Training	435	914	+479	87.5%
Validation	87	87	0	8.4%
Test	44	44	0	4.1%
Total	566	1045	+479	100%

**Table 4:** Defects found across the dataset.

Class Id	Class Name	Severity G	Ref. in Inspection Form	No. of Annotations
0	Corroded/oxidised reinforcement	5	ca_c.a.p._6	2259
1	Washed-out/deteriorated concrete	3	ca_c.a.p._3	545
2	Washed-out/deteriorated concrete at ends	4	ca_c.a.p._21	135
3	Crack	5	-	47
4	Protruding anchor bars	5	c.a.p._12	7
5	Cracks near stirrups	2	ca_c.a.p._15	260
6	Active moisture stains	3	ca_c.a.p._2	137
7	Passive moisture stains	1	ca_c.a.p._1	1023
8	Exposed/corroded stirrups	3	ca_c.a.p._16	897
10	Drainage streaks	3	Dif_Gen_1	550
11	Honeycombs	2	ca_c.a.p._4	215
Total				6086

**Table 5:** Performance metrics of the YOLO model.

Class Id	Severity	GT_TP	TP	FP	FN	Precision	Recall	F1 Score
0	5	41	29	9	12	0.76	0.71	0.73
1	3	27	17	9	10	0.65	0.63	0.64
2	4	7	5	2	2	0.71	0.71	0.71
3	5	2	2	0	0	1.00	1.00	1.00
4	5	2	2	0	1	1.00	0.67	0.80
5	2	10	6	4	4	0.60	0.60	0.60
6	3	11	7	3	4	0.70	0.64	0.67
7	1	31	21	9	10	0.70	0.68	0.69
8	3	25	15	8	10	0.65	0.60	0.63
10	3	27	17	9	10	0.65	0.63	0.64
11	2	9	5	3	4	0.63	0.56	0.59
					Mean	0.73	0.67	0.70

**Table 6:** Performance segmentation and object detection models.

Image Resolution [Mpx]	No. of Images	U-Net Segmentation Time (s)	SAM Segmentation Time (s)	YOLO Inference Time (s)
0.41 (640 × 640)	5	0.27	4.70	0.30
9.00 (4000 × 2250)	5	0.29	5.07	0.61
19.96 (5472 × 3648)	5	0.37	5.11	1.22

## Data Availability

Data will be made available on request.
